# A Highly Efficient and Durable Fluorescent Paper Produced from Bacterial Cellulose/Eu Complex and Cellulosic Fibers

**DOI:** 10.3390/nano9091322

**Published:** 2019-09-15

**Authors:** Mingquan Zhang, Xiao Wu, Zhenhua Hu, Zhouyang Xiang, Tao Song, Fachuang Lu

**Affiliations:** 1 State Key Laboratory of Pulp and Paper Engineering, South China University of Technology, Guangzhou 510640, China; fezhangmingquan@mail.scut.edu.cn (M.Z.); msxiaowu@mail.scut.edu.cn (X.W.); zhenhuahu1991@163.com (Z.H.); songt@scut.edu.cn (T.S.); 2 Guangdong Engineering Research Center for Green Fine Chemicals, Guangzhou 510640, China

**Keywords:** bacterial cellulose, Eu ion, complex, cellulosic fiber, fluorescent paper, durability

## Abstract

The general method of producing fluorescent paper by coating fluorescent substances onto paper base faces the problems of low efficiency and poor durability. Bacterial cellulose (BC) with its nanoporous structure can be used to stabilize fluorescent particles. In this study, we used a novel method to produce fluorescent paper by first making Eu/BC complex and then processing the complex and cellulosic fibers into composite paper sheets. For this composting method, BC can form very stable BC/Eu complex due to its nanoporous structure, while the plant-based cellulosic fibers reduce the cost and provide stiffness to the materials. The fluorescent paper demonstrated a great fluorescent property and efficiency. The ultraviolet absorbance or the fluorescent intensity of the Eu-BC fluorescent paper increased with the increase of Eu-BC content but remained little changed after Eu-BC content was higher than 5%. After folding 200 times, the fluorescence intensity of fluorescent paper decreased by only 0.7%, which suggested that the Eu-BC fluorescent paper has great stability and durability.

## 1. Introduction

Europium (Eu) element has one of the best photoluminescent properties among rare earth elements. Due to the 4f→4f transition, Eu^3+^ ions can convert ultraviolet light into visible light and have been widely used in fluoroimmunoassay [1,2]. The antenna effect induced by the chelation between Eu^3+^ and negatively charged ligand results in an even stronger photoluminescent performance for ligand/Eu^3+^ complexes. The complexes of various ligands, e.g., β-diketone, chelated with Eu^3+^ can be used as photoreceptors, superconductors, magnetic materials, catalysts, fluorescent probes, and light-emitting diodes [3,4,5,6,7,8].

Complexes of polymer ligands and rare earth elements are considered as high-efficiency light-emitting materials because of their strong photoluminescent ability, easy color mixing, low temperature sensitivity, and high thermal stability [9]. In the complex of cholesterol-g-poly(*N*-isopropylacrylamide) (PNIPAM) and Eu^3+^, the oxygen-nitrogen coordination between amide group and Eu^3+^ provides a strong fluorescent characteristic for Eu^3+^ [10]. Those ligands with functional groups containing strong electron-donating groups, e.g., hydroxyls, carboxyls, amides, and nitriles, especially have strong chelation effects with Eu^3+^ ions or other rare earth elements. Carboxymethyl cellulose (CMC) or cholesterol-g-poly(N-isopropylacrylamide) were used to complex with Eu or Tb, and they showed strong photofluorescent properties [11,12,13,14]. Hybridized nanofibers were produced between polyacrylonitrile and Eu^3+^, which had one-dimensional nanostructure and high specific surface area, showing excellent fluorescent properties [1]. However, most of these photofluorescent complexes or materials are still facing the problems of stability and durability, i.e., the fluorescence intensity decreases after severe use, due to the leaching or aggregation of fluorescent elements or particles.

When the polymer ligands reduce to nano- or submicron-scale, they provide the materials with special characteristics in mechanical, optical, thermal, or surface properties [15]. Nanocellulose, with its sustainable nature and nanoscale characteristics, inspires its application in functional materials. So far, only a few studies have used nanocellulose, e.g., cellulose nanocrystal (CNC), cellulose nanofibril (CNF), and bacterial cellulose (BC), as supports or ligands for fluorescent substances. Fluorescein-5′-isothiocyanate (FITC) was used to label CNCs for fluorescence bioassay and bio-imaging applications [16]. CNCs grafted by poly (N-isopropylacryalamide)(PNIPAAM) brushes had thermo-enhanced fluorescence and can be used in biomedical applications [17]. The silver nanocluster was loaded on natural CNFs, giving the material fluorescence properties and antimicrobial properties [18]. A fluorescent film was prepared by chemical modification of CNF film surface with boronate-terminated conjugated polymer chains and used to detect nitro-aromatic vapor [19].

Bacterial cellulose (BC) is a type of typical extracellular cellulose produced by acetobacter bacteria. BC was composed of ultrafine and interlaced nanofibers with a width of less than 100 nm to form a network structure. This structural feature provides BC with a large specific surface area and a nanoporous structure [20,21]. This nanoporous structure makes BC an ideal support for functional nanoparticles that can be further processed into functional materials. BC membranes were used to support TiO_2_ nanoparticles and blended with rare earth elements, e.g., lanthanum (La) and cerium (Ce), leading to a high photocatalytic efficiency [22]. BC and CdSe were combined to prepare composite films which have photoluminescence properties [23]. BC was used to support nitrogen-doped carbon nanofibers; this composite showed electrode material properties and was used to prepare a flexible all-solid-state high-power supercapacitor [24]. Pt was supported by BC to prepare a composite material having high catalytic efficiency for methanol fuel battery [25]. There are very few studies on the preparation of photofluorescent materials by using BC to support and form complexes with rare earth elements. The possible reason is the high cost of BC and its soft nature upon film forming, which is not able to provide a good stiffness for the materials.

Plant-based cellulosic fibers are well known to be the raw material for paper making and they can also be used as substrates or supporting materials for functional ligands to produce functional paper-based materials. Fluorescent paper has also been produced. Coating or printing method is a common way to composite the fluorescent materials with paper sheets. Fluorescent DNA-based oligodeoxyfluoroside dyes were printed on paper, which can sense food spoilage and ripening in the vapor phase [26]. 8-hydroxyquinoline aluminum (Alq3)-based bluish green fluorescent composite nanospheres were printed on paper as sensors for nitroaromatic explosive detection [27]. A ratiometric fluorescent probe-based paper sensor based on paper printed with gold nanoclusters stabilized by bovine serum albumin and fluorescent graphene oxide can be used for the determination of serum blood sugar [28]. Fluorescent inks such as heterorotaxane and the mixture of triethanolamine and UV absorbent UV-7282 were also used to print on paper for fluorescence [29,30]. Fluorescein isothiocyanate (FITC) and thioflavin were absorbed by nanocellulose and that was coated on the surface of paper to prepare fluorescent paper [31]. Most of the current methods produce fluorescent paper by attaching fluorescent materials to the surface of paper without altering the paper fiber structure. However, common cellulosic fibers do not have nanoporous structure and thus cannot well stabilize the nanosized fluorescent particles, leading to lack of stability, recycling efficiency, and durability.

BC can be tightly bound to cellulosic fibers due to their abundant surface hydroxyl groups [20,21], whose features can be used to prepare functional paper-based materials if BC is properly functionalized. In cellulosic fiber/BC composite functional materials, cellulosic fibers can reduce the cost of pure BC materials and provide high stiffness, while BC can still provide the materials with high toughness, high tearing, good stability, and durability. Catalytic paper sheets were produced by loading Pt or Au onto BC surface and then compositing with cellulosic fibers, which showed excellent catalytic efficiency, stability, and reusability [32,33]. In this study, we prepared fluorescent paper by making BC/Eu complex first and then processing the complex and cellulosic fibers into paper sheets. This method incorporates the fluorescent complexes as part of the cellulosic fiber matrix and thus may improve the stability and durability of fluorescent paper.

## 2. Materials and Methods

### 2.1. Materials

*Gluconacetobacter xylinus* ATCC23767 was obtained from Nanjing High Tech University Biological Technology Research Institute Co., Ltd. (Nanjing, China) and used to produce the bacterial cellulose (BC) pellicles according to the static fermentation method [20,21]. The degree of polymerization (DP) and Segal crystallinity index (CI) of BC was predetermined as 1081 and 96.0%, respectively [20,21].

Europium trioxide (Eu_2_O_3_), hydrochloric acid, and sodium hydroxide were purchased from Shanghai Macklin Biochemical Co., Ltd. (Shanghai, China). All other chemicals are analytical grade chemicals.

### 2.2. Preparation of EuCl_3_·6H_2_O

Eu_2_O_3_ (0.727 g) was completely dissolved in 6.0 mol/L HCl solution. The mixture was placed in boiling water bath for 30 min to evaporate the unreacted HCl. The solution was then diluted with deionized water to volume 100 mL to adjust the concentration to 0.0416 mol/L.

### 2.3. Preparation of Eu-BC

Approximately 20 g of wet BC pellicles (moisture content = 98.5%) was disintegrated by a lab homogenizer (SKG 1246, Foshan, China) for 20 s in 130 mL of water. The water was then filtered to obtain the disintegrated BC. The pH of the EuCl_3_·6H_2_O solution was adjusted to 5–6 with 1 M NaOH. The disintegrated BC was then added slowly to the EuCl_3_·6H_2_O solution at 45 °C under a magnetic stirring (400 rpm). The pH of mixture was adjusted to about 7 with 1 M NaOH. Finally, the mixture was refluxed at 70 °C for 1 h 30 min to prepare Eu-BC.

### 2.4. Manufacture of Fluorescent Paper

A vacuum filtering method was used to prepare the fluorescent paper because at high EuBC addition it would take a very long time at the water drainage step for the standard TAPPI method. In detail, bleached sugarcane bagasse pulps (BSBP) and Eu-BC were composited with different ratios, i.e., 1%, 2%, 5%, 10%, and 20% (the proportion of Eu-BC based on total paper dry weight). The mixture was homogenizer for 20 s and the mixture was filtered by a sand core filter device. After water was drained, the paper sheet was formed. Finally, the paper was dried in an oven at 9 °C for 50 min.

### 2.5. Characterization of Eu-BC and Fluorescent Paper

Nitrogen adsorption–desorption isotherm measurements were performed on an ASAP 2460 (Micromeritics, Norcross, GA, USA) volumetric adsorption analyzer at 77 K. The Brunauer–Emmett–Teller (BET) method was utilized to calculate the specific surface area of each sample. All samples were degassed at 120 °C at 0.05 atm for 6 h before the measurement began.

Fluorescent paper sheets or Eu-BC were taken under an AGL-9406 UV lamp for excitation at 256 nm. The ultraviolet light absorption of the samples was measured by solid-state ultraviolet spectrophotometer (UV-2450, SHIMADZU, Kyoto, Japan). The fluorescence spectra of the samples were recorded by a steady-state transient fluorescence spectrometer (FluoroMax-4, HORIBA Jobin Yvon, Longjumeau, France).

The Eu content of the paper was measured by an atomic absorption spectrometer (Z-2000, Hitachi, Tokyo, Japan). The surface morphologies of unmodified BC, Eu-BC, Eu-BC fluorescent papers were observed by a scanning electron microscope (SEM) (Merlin, Zeiss, Munich, Germany); X-ray photoelectron spectroscopy (XPS) was conducted on the fluorescent paper by a X-ray photoelectron spectrometer (Kratos Axis Ulra DLD, Kratos Analytical, Manchester, UK) to analyze the valence and binding energy of the Eu ions.

## 3. Results and Discussion

### 3.1. Characterization of Eu-BC and Fluorescent Paper

**Figure 1:**
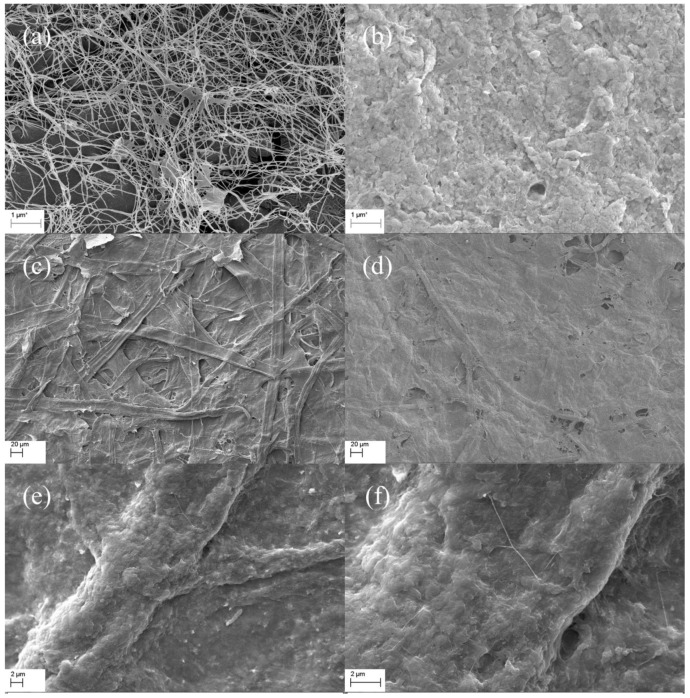
SEM images of (**a**) unmodified BC, (**b**) Eu-BC, (**c**) paper sheet made from sugarcane bagasse pulp, (**d**) Eu-BC fluorescent paper, and the cellulosic fibers on Eu-BC fluorescent paper at (**e**) 2K× magnification and at (**f**) 5K× magnification.

**Figure 2:**
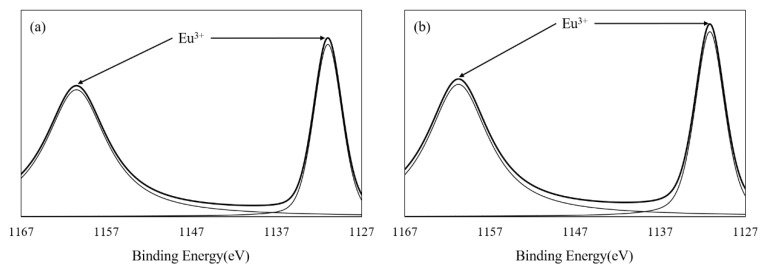
XPS spectra of (**a**) Eu-BC and (**b**) 20% Eu-BC fluorescent paper.

Bacterial cellulose (BC) is a natural cellulosic material with nanoporous structure, which provides the advantages to stably support functional nanoparticles. The BC used in this study was prepared by static fermentation method instead of agitated method because BC prepared by static method provides paper with better mechanical properties [21]. The BC had a specific surface area of 41.69 m^2^/g and a pore volume of 0.1598 cm^3^/g (Table 1). As can be seen from the SEM image (Figure 1a), BC is composed of ultrafine and reticular nanofibrils demonstrating a large specific surface area. Eu-BC complex was prepared by adsorbing Eu^3+^ onto the nanoporous structure of BC. The total Eu content of Eu-BC was 32.3 wt% (Table 1), which suggested that the Eu ions were successfully adsorbed onto BC. The XPS spectrum of Eu-BC showed that the binding energies of the two peaks were close to 1126 and 1155 eV, which belonged to the Eu^3+^ valence state and suggested that all the Eu in BC was still in Eu^3+^ forms (Figure 2a). The XPS spectrum indicated that Eu^3+^ ions were successfully coordinated with BC to form a Eu-BC complex. Specific surface area and pore volume of Eu-BC decreased greatly to 6.84 m^2^/g and 0.0214 cm^3^/g, indicating Eu^3+^ formed uniform complex with BC and occupied most of pores on BC surface. As demonstrated by SEM image (Figure 1b), Eu-BC complex was evenly distributed or dispersed among the BC matrix. Eu-BC complex was evenly dispersed because of the nanoporous structure of BC, and it also indicated the formation of nanosized complex particles.

**Figure 3:**
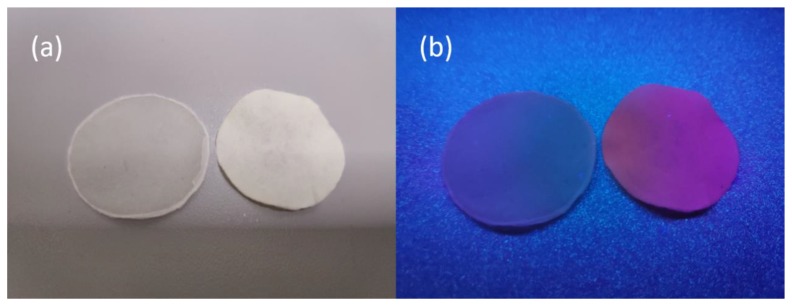
Photographs of 20% BC paper (without Eu) and 20% Eu-BC fluorescent paper (**a**) under visible light and (**b**) under ultraviolet light.

The Bleached sugarcane bagasse pulp (BSBP) was composited with Eu-BC to produce fluorescent papers with different Eu-BC contents. The Eu content in Eu-BC fluorescent paper as determined by atomic absorption spectrometry was proportional to the amount of Eu-BC added to the production of the fluorescent paper, demonstrating a uniform adsorption of Eu ions onto BC (Table 1). Most of the Eu elements in the fluorescent paper were still in the valence state of 3+ (Figure 2b), facilitating the photofluorescence of the fluorescent paper. The fluorescent paper showed red fluorescence under ultraviolet light irradiation at 256 nm (Figure 3), indicating that the energy of ultraviolet light absorbed is effectively transferred to Eu^3+^ through the “antenna effect”, making the fluorescent paper glow red [11,12,13,14]. It can be seen from the SEM images that almost all the bagasse fibers were uniformly covered by Eu-BC (Figure 1c–e). The reticular structure of BC can also be identified on fiber surface (Figure 1f).

### 3.2. Photofluorescent Properties of Eu-BC and Eu-BC Paper

**Figure 4:**
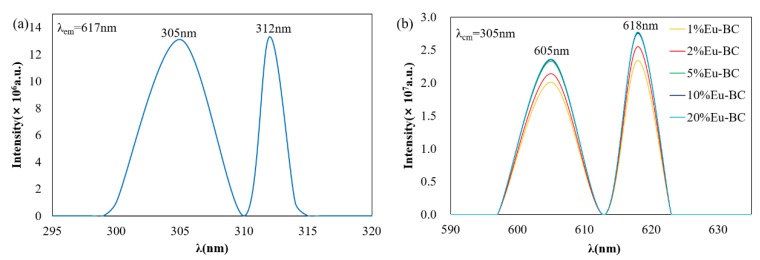
(**a**) Fluorescence excitation spectra of Eu-BC and (**b**) fluorescence emission spectra of fluorescent paper sheets with different Eu-BC contents.

As can be seen from Figure 4, there were two peaks in the Eu-BC excitation spectrum, 305 nm and 312 nm (Figure 4a). The 305 nm wavelength was selected to excite fluorescent papers of different Eu-BC contents to obtain a fluorescence emission spectrum. With the excitation at 305 nm wavelength, the fluorescent paper produced radiation emission at 605 nm and 618 nm (Figure 4b), which should correspond to Eu^3+^ magnetic dipole transition of ^5^D_0_→^7^F_1_ and electric dipole transition of ^5^D_0_→^7^F_2_ [1]. The peaks of Figure 4b suggested that the emission intensity of fluorescent paper below 5% Eu-BC content increases with the increases of Eu-BC content. The emission intensity of fluorescent paper conducted no more increases when the EU-BC content in fluorescent paper was greater than 5%.

**Figure 5:**
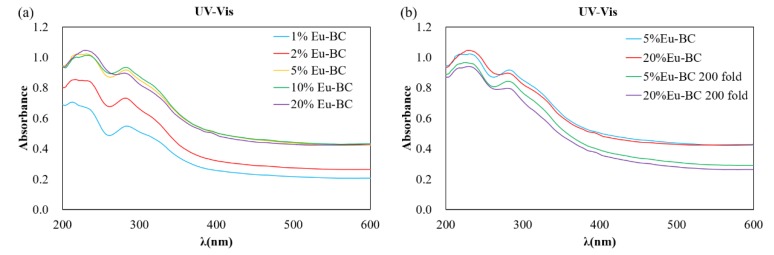
(**a**) UV-Vis absorbance spectra of fluorescent paper sheets with different Eu-BC contents and (**b**) changes in UV-Vis spectra after fluorescent paper was folded 200 times.

By evaluating the solid-state ultraviolet absorption spectrum (Figure 5a), the fluorescent paper had two absorbance peaks at the wavelength of ~230 and ~280 nm. The absorbance of 1%, 2%, and 5% Eu-BC fluorescent paper successively increased with the increase of Eu-BC content. However, the absorbance of 5%, 10%, and 20% Eu-BC fluorescent paper remained almost unchanged. This result was consistent with the fluorescence excitation spectrum, showing that after Eu-BC content exceeds 5% the fluorescent property would not change for the fluorescent paper.

**Figure 6:**
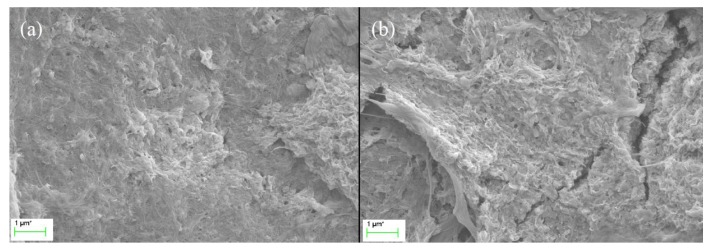
SEM images of (**a**) 5% Eu-BC fluorescent paper and (**b**) 20% Eu-BC fluorescent paper at 10K× magnification.

The structure of 5% Eu-BC and 20% Eu-BC fluorescent paper were shown in Figure 6. For the 5% Eu-BC paper, the reticular structure of BC can still be seen, but for the 20% Eu-BC paper, the paper was all covered with Eu-BC complex packed full with Eu elements, in which some Eu-BC complexes were observed to be stacked with each other. Many Eu-BC complexes or Eu particles were squeezed or buried inside the middle of the composite, which made them hard to be stimulated by ultraviolet light. This statement can be proved by the surface area, pore volume, and pore size data of EuBC paper (Table 1). For 1% EuBC paper, the cellulosic fibers were not entirely covered with EuBC, so it had relatively high pore volume and pore size; for 5% EuBC paper, the cellulosic fibers were largely covered with EuBC, so its pore volume and pore size decreased; for 20% EuBC paper, many EuBC stacked together, building some now pores between layers leading to the increase in pore volume and pore size (Table 1). Similar results were also found in the studies of poly(acrylonitrile)/Eu^3+^ complex that high Eu^3+^ does not necessary lead to improved emission intensity due to the stacking of emission centers [1]. This explains the fluorescence intensity or ultraviolet absorbance remaining constant when Eu-BC is over 5%. It also indicated that 5% is the most economical amount for Eu-BC to composite with cellulosic fibers.

### 3.3. Stability and Durability of Fluorescent Paper

**Figure 7:**
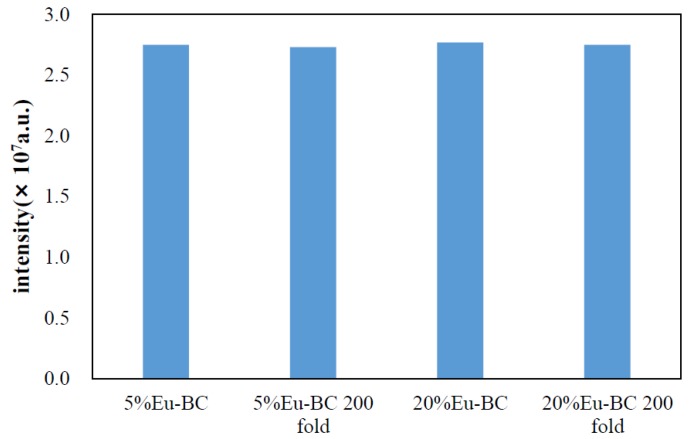
Changes of fluorescence emission spectra at 618 nm for Eu-BC fluorescent paper after 200 foldings.

To investigate the stability and durability of fluorescent paper, 5% Eu-BC fluorescent paper and 20% Eu-BC fluorescent paper were repeatedly folded 200 times. From the ultraviolet absorbance spectrum in Figure 5b, it can be found that the absorbance peaks decreased only slightly after folding 200 times. The absorbance of 5% Eu-BC fluorescent paper decreased by only 0.05 after 200 folds, while the 20% Eu-BC paper decreased by nearly 0.1. The fluorescence intensity at 618 nm emission changes after the folding of the fluorescent paper were shown in Figure 7. The intensity of the fluorescent paper only decreased by ~0.7% after 200 folds. This result suggested that the fluorescent paper has great stability and durability, and 5% Eu-BC fluorescent paper is even better than the 20% Eu-BC fluorescent paper. It suggested that the nanoporous and reticular structure of BC can help to form very stable complex with Eu ions, by preventing the Eu particles from leaching or aggregating.

### 3.4. Comparison of Eu-BC Fluorescent Paper with Other Composite Fluorescent Materials

We mainly compared the fluorescence intensity of different substrates complexing with Eu^3+^ ions, such as CMC [12], polydimethylsiloxane (PDMS) [34], β-diketone,4-imidazol-4,4,4-trifluorobutane-1,3-dione(HIDTFBD) [3], 2-(4′4′4′-trifluoro-1′3′-dioxobutyl)-carbazole (2-TFDBC) [35], and 2,7-bis(4′4′4′-trifluoro-1′3′-dioxobutyl)-carbazole (2,7-BTFDBC) [35]. The highest fluorescent emission intensity of the Eu-BC fluorescent paper in this study can reach 2.77 × 10^7^ a.u., showing higher value than many of the Eu-ligand complex fluorescent materials; the Eu-BC fluorescent paper also had a lowest Eu mass ratio among all showing its high efficiency (Table 2). This may be due to the high surface area of BC providing large numbers of chelating centers for Eu^3+^ and the nanoporous structure of BC providing uniform distribution of Eu elements. In addition, our Eu-BC fluorescent paper maintained good fluorescence performance after 200 folds. These comparisons with similar studies demonstrate the high efficiency and great durability for our Eu-BC fluorescent paper.

## 4. Conclusions

Fluorescent paper was prepared by compositing the Eu-BC complex with sugarcane bagasse pulps (BSBP). The fluorescent paper demonstrated a great fluorescent property and efficiency, i.e., having very low Eu mass content but high fluorescent intensity, compared to many of its counterpart fluorescent materials based on Eu/ligand complex. The ultraviolet absorbance or the fluorescent intensity of the Eu-BC fluorescent paper increased with the increase of Eu-BC content but remained little changed after Eu-BC content was higher than 5%, which was due to excessive Eu-BC complexes stacking with each other without effective irritation by ultraviolet light. After 200 times of folding, the fluorescence intensity of fluorescent paper decreased by only 0.7%, which suggested that the Eu-BC fluorescent paper has very good stability and durability. The high durability of the fluorescent paper can be attributed to the nanoporous or reticular structure of BC stabilizing the Eu particles.

## Figures and Tables

**Table 1:** Eu contents and BET parameters of BC, Eu-BC, and fluorescent paper sheets with different Eu-BC contents.

Sample	Eu wt%	Specific Surface Area (m^2^/g)	Pore Volumn (cm^3^/g)	Average Pore Radius (nm)
BC	0	41.69	0.1598	15.81
Eu-BC	32.30	6.84	0.0214	15.62
1% Eu-BC fluorescent paper	0.32	6.81	0.0991	79.84
2% Eu-BC fluorescent paper	0.88	-	-	-
5% Eu-BC fluorescent paper	1.53	3.17	0.0024	5.81
10% Eu-BC fluorescent paper	2.44	-	-	-
20% Eu-BC fluorescent paper	7.74	12.12	0.0430	14.74

**Table 2:** Comparison of composites with different substrate loadings of Eu^3+^ ions.

Base	Reaction Temperature (°C)	Reaction Time (h)	Eu Mass Percentage	Fluorescent Emission Intensity (a.u.)	Reference
BC/Paper	70	1.5	1.5%	2.77 × 10^7^	this work
CMC	70	0.25	4.4%	1.05 × 10^7^	[12]
HIDTFBD	60	6	18.0%	4.21 × 10^6^	[3]
PDMS	60	3	2%	1.42 × 10^4^	[34]
2-TFDBC	60	6	16.7%	6.90 × 10^6^	[35]
2,7-BTFDBC	60	6	24.4%	6.20 × 10^6^	[35]
